# Mapping the Access of Future Doctors to Health Information Technologies Training in the European Union: Cross-Sectional Descriptive Study

**DOI:** 10.2196/14086

**Published:** 2019-08-12

**Authors:** Guido Giunti, Estefania Guisado-Fernandez, Hrvoje Belani, Juan R Lacalle-Remigio

**Affiliations:** 1 University of Oulu Oulu Finland; 2 University College Dublin School of Public Health, Physiotherapy and Sports Science Dublin Ireland; 3 Insight Centre for Data Analytics Dublin Ireland; 4 Ministry of Health Zagreb Croatia; 5 Universidad de Sevilla Seville Spain

**Keywords:** medical informatics, health information technologies, medical education, European Union

## Abstract

**Background:**

Health information technologies (HITs) such as electronic health records (EHR) and telemedicine services are currently used to assist clinicians provide care to patients. There are many barriers to HIT adoption, including mismatches between investments and benefits, disruptions in the workflow, and concerns about privacy and confidentiality. The lack of HIT training of health professionals as a workforce is an increasingly recognized and understudied barrier.

**Objective:**

The purpose of this study is to describe what courses on HIT topics are available at the graduate level for future health professionals in the European Union (EU) and to explore possible determining factors for their exposure to these courses.

**Methods:**

A cross-sectional descriptive study of EU medical schools was performed to explore the prevalence of HIT courses. The curricula of all identified higher learning institutions that offer a medical degree were manually explored to identify graduate-level courses that offer specific training on HIT topics. HIT topics were defined as courses or subjects that provided knowledge on the design, development, use, and implementation of HIT. Associations among potential factors such as population, yearly medical graduates, total number of physicians, EHR presence, and gross domestic product (GDP) were explored.

**Results:**

A total of 302 medical schools from the 28 member states of the EU were explored. Only about one-third (90/302, 29.80%) of all medical degree curricula offered any kind of HIT course at the graduate level; in the medical schools that offered HIT courses, the courses were often mandatory (58/90, 64.44%). In most EU countries, HIT courses are offered in less than half of the medical schools, regardless of the country’s GDP per capita. Countries with the highest percentages of HIT course presence have the lowest GDP per capita. There seems to be a weak inverse correlation (–0.49) between the two variables (GDP per capita and HIT course presence). There is a trend between the availability of medical human resources and an increase in the presence of HIT courses, with Romania, Croatia, and Greece as outliers in this respect.

**Conclusions:**

The current state of medical training in the EU leaves much room for improvement. Further studies are required for in-depth analysis of the content and manner of instruction that would fit present and future needs of HIT.

## Introduction

Health information technologies (HITs) [1] are used in health care institutions to assist clinicians provide care with tools such as electronic health records (EHR) and telemedicine services [2]. The increasingly vast amounts of health data available provide health professionals access to new ways to collect, analyze, and use that information [3,4]. The use of EHRs for documentation allows data to be organized around diseases or quality indicators, machine learning and artificial intelligence enable population health analytics to identify predictive characteristics and factors for diseases, and remote tools facilitate disease management in point-of-care and home settings [5].

There are many barriers to HIT adoption, including mismatches between investments and benefits, disruptions in the workflow, and concerns about privacy and confidentiality [6-8]. However, while many health professionals and students see the potential benefit of these technologies in health care, many are also frustrated [9,10], struggling to adapt, without knowing the underlying science of information in these new tools. The lack of HIT training of health professionals as a workforce is an increasingly recognized and understudied barrier [4,11,12].

Focused and concerted educational training in health informatics is essential for health professionals to realize the full benefit of the data and tools that are already part of the practice of medicine and to help develop new and improved tools of the future. There are some initiatives worldwide to provide specialization training on medical informatics, such as the American Medical Informatics Association in the United States, with nearly 1700 board certified professionals [13], or England’s efforts with the Topol review [14]; many other countries have not, or at least not sufficiently, established such opportunities until now [4]. A recent publication in the British Medical Journal proposes that literacy in informatics should be a formal requirement of all medical education, biomedical research, and public health training [15].

The current state of how future doctors are trained in HIT contents is largely unknown in many European Union (EU) countries. Further, although there may be opportunities for obtaining education in this field, most are targeted at the postgraduate level, leaving HIT training to be pursued as a professional interest and not a core skill. The purpose of this study is to describe what courses on HIT topics are available at the graduate level for future health professionals in the EU and to explore possible determining factors for their exposure to these courses.

## Methods

### Study Design

A cross-sectional descriptive study of the state of EU medical education was performed to explore the prevalence of specific HIT courses offered to future doctors. Cross-sectional studies are carried out at one time point or over a short period to estimate the prevalence of the outcome of interest for a given population [16].

The curricula of all identified higher learning institutions such as faculties of medicine, schools of medicine, or universities that offer the medical degree, hereon referred to as “medical schools,” were explored to identify graduate-level courses that may offer would-be physicians training on any HIT topics. Potential factors such as population, yearly medical graduates, total number of physicians, EHR presence, and GDP were explored for associations.

### Settings

The EU is a political and economic union of 28 member states, with an estimated population of over 513 million and almost 2 million practicing physicians [17]. During the first quarter of 2018, the medical degree programs from all EU member states were systematically explored and classified.

### Data Sources

No official list of medical schools in EU exists; therefore, a preliminary list was obtained and refined from gray literature [18]. Multimedia Appendix 1 shows the definitive list of medical schools used.

Data on EU population statistics, GDP per capita, EHR presence, and medical graduates and doctors were extracted from their respective official sources [17,19,20]. Latest available information was used, and cases where no information was available were marked.

### Selection Criteria

For the purpose of this study, and given the many different terms in use in the field, the terms medical informatics, health information and communications technology (ICT), eHealth, mHealth, biomedical and health informatics, consumer health informatics, digital health, and other variations were considered to be encapsulated by the term “HIT.”

HIT topics were defined as courses or subjects that provided knowledge on the design, development, use, and implementation of HIT. The different course names and programs (where available) were revised for mentions of HIT contents. Courses that used ICT tools to enhance medical education on other subjects (eg, anatomy lessons or clinical case virtual simulations), or who only taught ICT content as a means to an end (eg, programming in R for biostatistics) were not considered to be focused on HIT training and were therefore not included.

A small random sample (10%) was independently reviewed to assess clarity of the selection criteria, and interrater reliability was calculated using the Fleiss-Cohen Coefficient. Once this was established, the remaining data were explored. Disagreements were resolved by consensus involving a third reviewer, when necessary.

### Data Extraction and Classification

Medical education data were manually extracted from each medical school’s website to obtain the latest publicly available curricula and program. A multilingual and multicultural team (GG, EG, and HB) conducted the data extraction, and any language barrier issue was resolved using the Google Translate feature. The team independently reviewed and classified the extracted information using structured forms. In Multimedia Appendix 2, the raw data are presented.

### Statistical Methods

Categorical variables are presented as absolute and relative frequencies. Quantitative variables are presented as mean and SD or median and interquartile range, depending on the distribution. The Landis and Koch standards for the Fleiss-Cohen coefficient were used [21]. Statistical analysis was performed using R (Vienna, Austria, R Foundation for Statistical Computing; 2013).

## Results

Interrater reliability was determined using the Cohen kappa statistic by extracting a random sample (n=32) and independently reviewing the selection criteria. Kappa was found to be more than acceptable at 0.91 (SE 0.06, 95% CI 0.79-1.0).

**Table 1:** Summary of the presence of health information technology courses in each European Union member state.

Country	Total medical schools (N=302), n (%)	Medical schools with HIT^a^ courses (n=90), n (%)	Medical schools with HIT courses, where the course is mandatory (n=58), n (%)	EHR^b^ availability
Austria	6 (2.0)	1 (16.67)	1 (100)	Nationwide project in progress
Belgium	10 (3.3)	1 (10.00)	0 (0)	Nationwide project in progress
Bulgaria	6 (2.0)	2 (33.33)	1 (50)	Yes
Croatia	4 (1.3)	3 (75.00)	3 (100)	Nationwide project in progress
Cyprus	4 (1.3)	1 (25.00)	1 (100)	Nationwide project in progress
Czech Republic	9 (2.9)	3 (33.33)	2 (66.67)	Nationwide project in progress
Denmark	4 (1.3)	0 (0.00)	N/A^c^	Yes
Estonia	1 (0.3)	0 (0.00)	N/A	Yes
Finland	5 (1.7)	1 (20.00)	1 (100)	Yes
France	34 (11.26)	10 (29.41)	7 (70)	Nationwide project in progress
Germany	38 (12.6)	16 (42.11)	8 (50)	Nationwide project in progress
Greece	7 (2.3)	6 (85.71)	5 (83.33)	Nationwide project in progress
Hungary	4 (1.3)	1 (25.00)	1 (100)	Yes
Ireland	6 (2.0)	2 (33.33)	1 (50)	Nationwide project in progress
Italy	41 (13.6)	7 (17.07)	7 (100)	Nationwide project in progress
Latvia	2 (0.7)	0 (0)	N/A	Nationwide project in progress
Lithuania	2 (0.7)	0 (0)	N/A	Yes
Luxembourg	1 (0.3)	0 (0)	N/A	Nationwide project in progress
Malta	1 (0.3)	0 (0)	N/A	Yes
Netherlands	9 (3.0)	3 (33.33)	0 (0)	Nationwide project in progress
Poland	19 (6.3)	7 (36.84)	7 (100)	Nationwide project in progress
Portugal	7 (2.3)	2 (28.57)	1 (50)	Nationwide project in progress
Romania^d^	13 (4.3)	10 (76.92)	5 (50)	Nationwide project in progress
Slovakia^d^	3 (1.0)	0 (0)	0 (0)	Nationwide project in progress
Slovenia	2 (0.7)	2 (100)	1 (50)	Nationwide project in progress
Spain	25 (8.3)	8 (32)	4 (50)	Nationwide project in progress
Sweden	7 (2.3)	0 (0)	N/A	Yes
United Kingdom	32 (10.6)	2 (6.25)	2 (100)	Yes

^a^HIT: health information technology.

^b^EHR: electronic health record.

^c^N/A: not available.

^d^Information on the medical school curricula in one school each in Romania and Slovakia was not available.

A total of 302 medical schools from the 28 member states of the EU were explored. Only one-third (90/302, 29.80%) of all medical degree curricula offered any kind of HIT course on the graduate level; in medical schools that offered HIT courses, the courses were often mandatory (58/90, 64.44%). The prevalence of HIT courses for medical degree students in EU member states is very low. As an indicator of system-wide informatization, we also compared our findings with reports and literature on each EU country’s process of EHR implementation. The state of EHR implantation in the EU is a work in progress in most cases (only one-third have nationwide systems). Table 1 presents the information according to each country.

**Figure 1:**
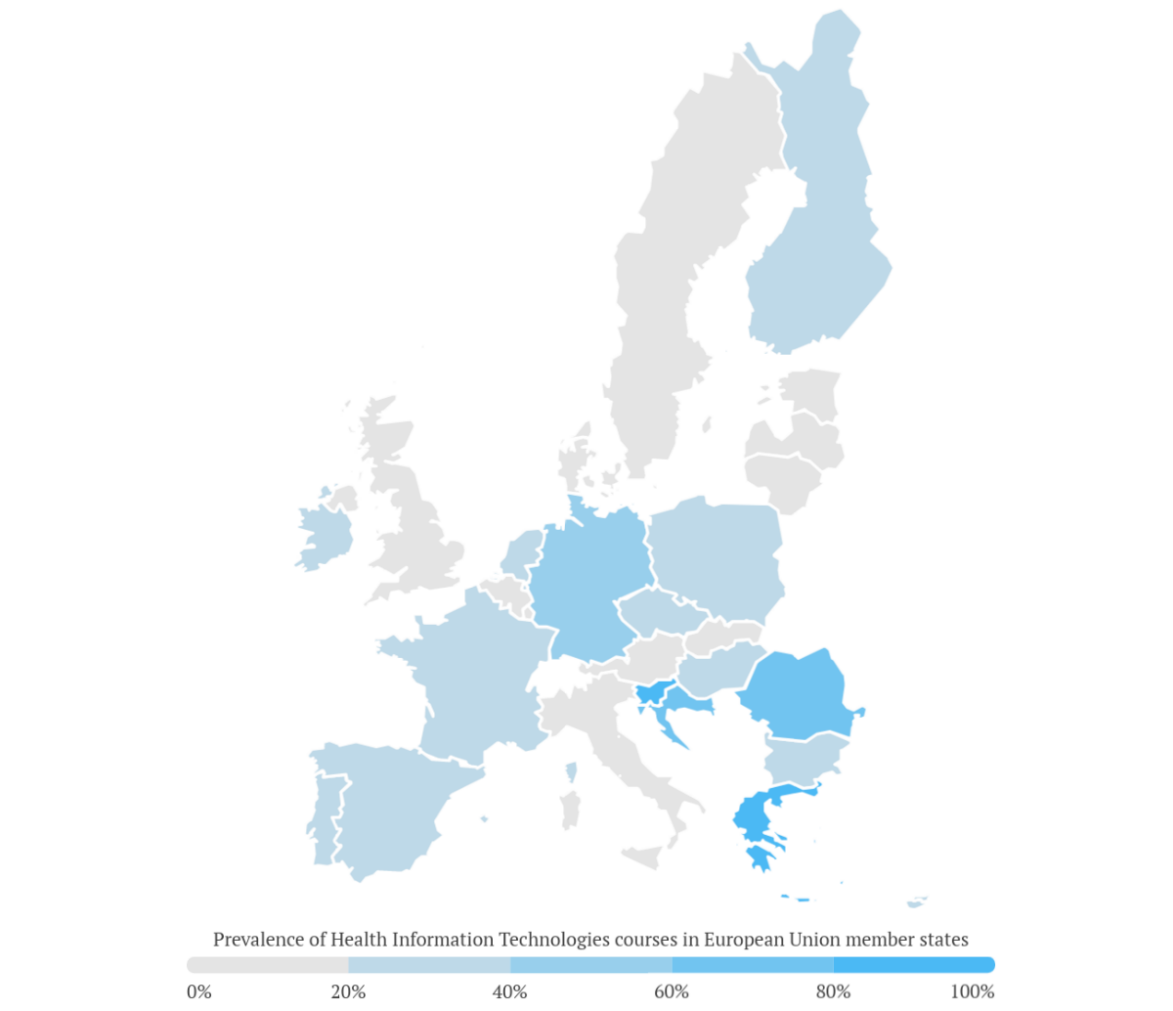
Presence of health information technology courses in the European Union member states.

HIT courses offered in most medical schools were titled along the lines of “Medical informatics,” “Telemedicine,” “e-Health,” or “Health informatics” and usually paired with statistics content. Some universities also provided courses on relatively advanced topics such as “AI in Medicine” (Medical University of Lublin, Poland), “Robotics programming with LEGO” (University of Duisburg-Essen, Germany), or workshops on “Application scenarios of Virtual Reality” (University of Ulm, Germany). Entrepreneurship was related to HIT in some cases, for example, in the Galway School of Medicine (National University of Ireland, Ireland), courses on “Becoming a Medical Innovator” are offered. Figure 1 shows a map with EU member states and the presence of HIT courses. A list of all the course names and their frequency can be found in Table 2.

**Table 2:** List of all the names of health information technology courses and their frequencies.

Course name	Frequency
Medical informatics	22
Epidemiology, medical biometry and medical informatics	10
Medical informatics and biostatistics	9
Introduction to Medical Informatics	7
Information Technologies	5
Research and New Technologies	3
Biomedical Information Systems	2
Evaluation of methods of analysis applied to life and health sciences	2
Medical IT^a^	2
Advanced Medical Technology	1
Basics of informatics in the health sector	1
Basics of Medical Informatics	1
Becoming a Medical Innovator	1
Bioinformatics	1
Biomedical Research and New Technologies	1
Biometrics and Epidemiology	1
Clinical Informatics and Biostatistics	1
eHealth^b^	1
eHealth & Medical Informatics	1
Health informatics	1
Health Technology Assessment	1
Healthcare Imaging and Information Systems,	1
ICT^c^ for Medicine	1
Informatics and Applications of Medical Informatics	1
IT Resources; Telemedicine	1
Lecture Epidemiology and Medical Informatics	1
Legal and Organisational Aspects of Medicine - includes Health IT	1
Medical and Scientific Methodology (includes Bioengineering and Medical Informatics)	1
Medical Applied Informatics	1
Medical Computer Science	1
Medical Informatics and Internet Computer Certificate	1
Medical Informatics, Biomedical Statistics, and scientific English	1
Medical informatics, e-Health^b^ and medical statistics	1
Medicine and Technology	1
Modern Informatics in Biomedicine	1
New technologies in biomedicine	1
Robotics and Programming with Lego - An introductory Course to Robotics and Programming for Medical Students	1
Statistics and Bioinformatics	1
Tele-Health & Health Information Technologies In Public Health	1
Telemedicine and eHealth	1
Telemedicine: internet technologies for health	1
Telemedicine: Possibilities and Limitations	1
The Industry Perspectives on Innovative Medicine Intensive Summer Course	1

^a^IT: information technology.

^b^eHealth/e-Health: electronic health.

^c^ICT: information and communication technology.

**Figure 2:**
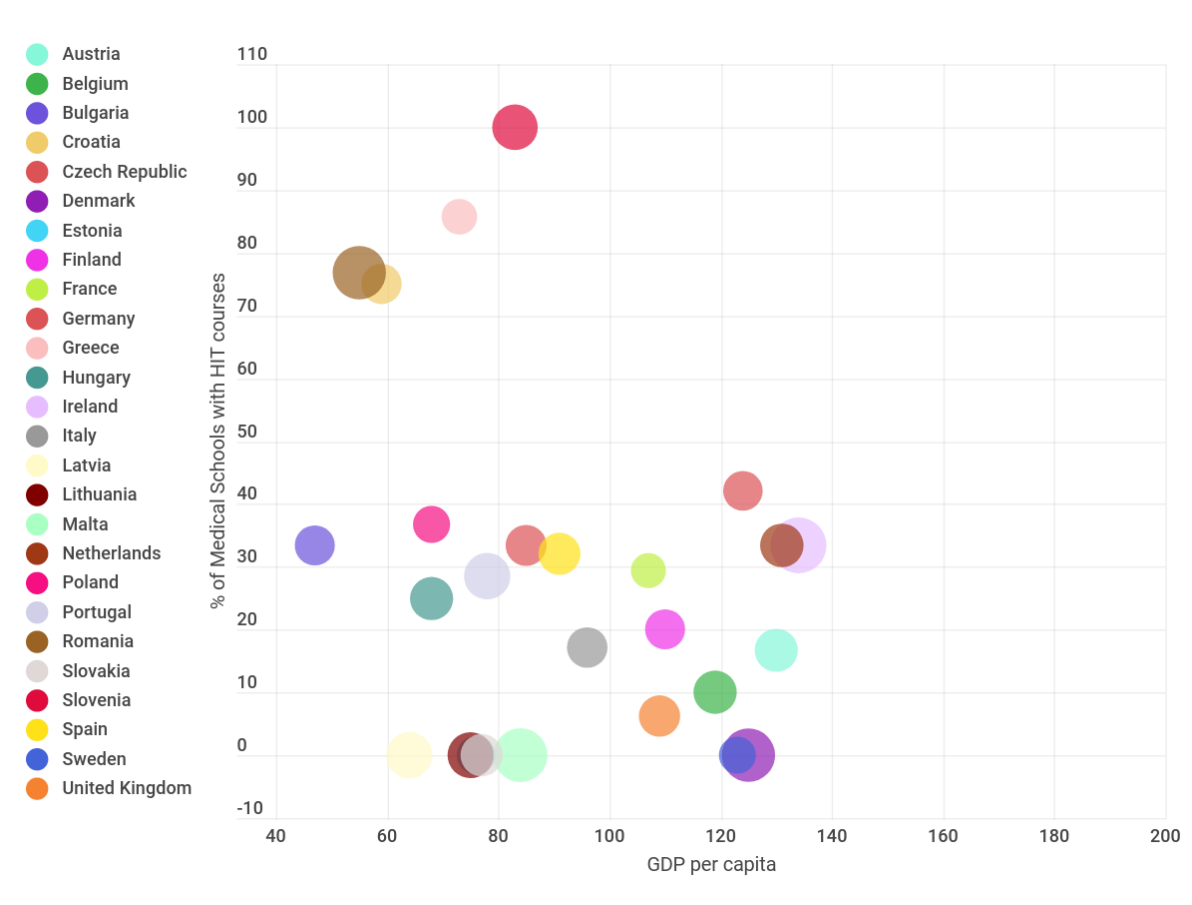
Relationship between European Union member states' GDP per capita and presence of HIT courses. The surface represents the number of medical graduates per year over 100,000 inhabitants. Countries where no information on yearly medical graduates was available (Cyprus and Luxembourg) are not shown. GDP: gross domestic product; HIT: health information technology.

We also explored possible associations between the presence of HIT courses and EU population statistics, GDP per capita, EHR presence, and yearly new medical graduates per country. Most countries seem to offer HIT courses in less than half of their medical schools, regardless of their GDP per capita. Countries with the highest percentages of HIT course offerings are among the ones with the lowest GDP per capita in EU. There seems to be a weak inverse correlation (–0.49) between the two variables (GDP per capita and HIT course offer). In Figure 2, we show a scatter plot with the relationship between GDP per capita and percentage of medical schools with HIT courses. The exploration of population size, the main demographic variable, resulted in no relation between the variables.

**Figure 3:**
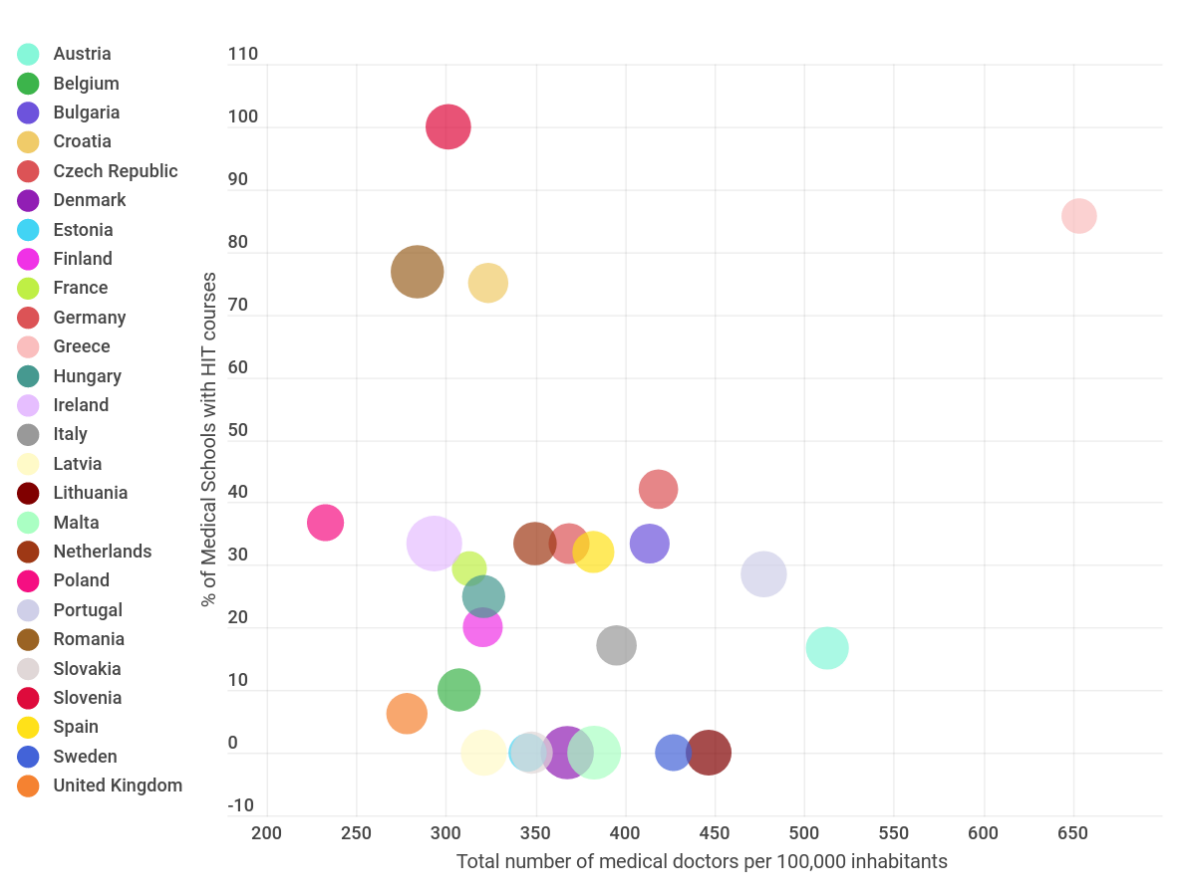
Relationship between the total number of physicians per European Union member state and the presence of HIT courses. Countries where information was missing (Cyprus and Luxembourg) are not shown. HIT: health information technology.

We explored the association of a country’s population over the number of physicians and the presence of HIT course offerings. There seems to be a trend between the availability of medical human resources and an increase in the presence of HIT courses, with Romania, Croatia, and Greece as outliers (Figure 3).

## Discussion

### Principal Findings

This work provides an overview of the state of HIT courses available for graduate-level medical students in the EU. It offers unique insight into the prevalence of HIT courses for each country and explores possible associated factors. Medical students in the EU seem to find it difficult to gain exposure to HIT courses, with only one-third of all medical schools offering any courses on these topics at all. The uneven distribution of training opportunities for medical students on HIT contents between the different countries highlights the need of further work on improving the medical curricula.

### Comparison With Prior Work

Despite their promise, HITs have proven difficult to implement [22], and there seems to be a failure to achieve widespread understanding of the benefits of technologies such as electronic record keeping and information exchange [23]. Although technology is increasingly being used in health care settings, health care professionals remain reticent to adopting HIT at different levels [24].

The need to improve the HIT competences of the health care workforce has been frequently emphasized by policymakers at an international levels [25]. One of the projects aiming to identify health care workforce IT skill needs is the CAMEI (Coordination Actions in the scientific era of Medical Education Informatics) project, which is a collaboration between the United States and Europe [26]. Further, the importance of training for the health care workforce in the use of new technologies was also acknowledged in many studies [25,26]. Several authors have pointed out that the training and competences that health care professionals receive regarding ICTs, in general, and as HIT end users, in particular, act as key factors in HIT adoption [27]. The limited exposure that medical students in the EU receive on HIT contents can easily explain the resistance that HIT implementations face later in health services settings. Recommendations from the International Medical Informatics Association [4] suggest that education on HIT contents should be available to all types of health care professionals, regardless of the types of specialization and levels of education as a way of emphasizing the formal penetration of the discipline and its concepts.

A significant factor for the slow uptake of HIT for health care professionals also seems to be the lack of customization that software vendors offer for their solutions [28,29]. The concept of user-centered design places the needs and characteristics of end users at the center of software design [30-32]. However, involving stakeholders in the design and development of technologies is not yet common practice for HIT solutions.

The introduction of HIT should not be viewed as a problem in technology exclusively, but rather as a problem in organizational change [33]. The implementation of HIT in the different EU member states is very diverse, particularly regarding the development of EHRs [27], a central component of an integrated HIT [34]. This heterogeneity seems to also apply to how health professionals are trained at the graduate level with regard to HIT concepts, with countries ranging from no training at all offered to all medical schools requiring the approval of HIT courses to obtain an medical degree.

In addition, it is known that health care spending per capita is positively correlated with GDP per capita [35] and that the use of health information systems may be a method to control increasing health spending [35]. Although more data would be needed to conclude a definitive casual relation, an interesting finding of our study was a weak inverse relationship between the presence of HIT courses and a country’s GDP. It would be interesting to ascertain what other factors and how the design of medical program curricula can impact a country’s health care expenditure.

Many factors that determine how a degree’s curriculum is structured, and while there is a need for change, political factors that prevent much needed modifications may exist. It is important to remember that wide-scale implementation of EHRs and other HIT systems has often been the result of state or national legislations [19]. Initiatives like these exist in many EU countries, but they are relatively recent and the change to curricula takes time. For example, digital competence has been acknowledged as one of the eight key competences for lifelong learning by the EU [36]. Under this framework, governments should encourage universities, particularly medical schools, to introduce formal teaching activities within undergraduate medical studies.

### Limitations

The findings of this study should be interpreted in the context of its limitations. The main limitation of this study is that it focuses on courses that specifically teach HIT contents and does not account for how ICTs can be used in different teaching methods such as problem-based learning, or other subjects where HIT concepts may be taught indirectly. Additionally, this study did not perform an in-depth analysis of the medical degree curricula; therefore, it is possible that HIT lessons are included or integrated in other courses. However, given that medical education is usually divided in preclinical studies (eg, basic sciences such as anatomy, physiology, and biochemistry) and clinical studies (eg, various areas of clinical medicine such as internal medicine, pediatrics, obstetrics, and gynecology), it is unlikely that foundational HIT contents would be found within these modules.

Analysis and interpretation was performed using the retrieved information. Newer data could be available through internal channels and publications of each institution or country. Nevertheless, our study used publicly available data, which would also be available to other researchers.

In recent years, new bachelor and master degrees of an interdisciplinary nature have been developed (eg, biomedical engineering). Universities that hold these degrees may have contents or courses available for medical degree students to access; however, as these courses would not appear in the medical degree curricula, these were not explored or included. Further, this study only focuses on medical degree students and does not cover other health care professionals (eg, nurses or physiotherapists) or disciplines that could have some overlap (eg, biomedical engineering).

Finally, an interplay of varying complex factors determines how a degree program and curriculum are designed, both within and outside academic institutions such as local, national, and regional needs; legislations and ruling legal frameworks; and political leadership and visions. These were not included, as they would be too many and exceed the scope of this study.

### Conclusions

Technology is transforming health care by slowly changing the way medicine is practiced. As HITs become more prevalent, expectations for health professionals to be fluent users of health technologies will continue to increase. The current state of medical training in the EU in this regard leaves much room for improvement. Further studies are required for in-depth analysis on the type of contents, proper placement in the medical curricula, and manner of instruction that would fit present and future needs of HIT.

Taking into consideration that the use of HIT is likely to continue to increase even further in most European health care systems, the need of future physicians to be literate in both medical sciences and HIT will soon no longer be optional. Empowering the following generations of doctors to allow them to take advantage of the full benefits that technologies have to offer is ever more important. A thorough look at the way we design our medical school curricula is needed.

## Appendix

Multimedia Appendix 1 List of medical schools in the EU.

Multimedia Appendix 2 Data on medical schools.

## Abbreviations

CAMEI: Coordination Actions in the scientific era of Medical Education Informatics
eHealth: electronic health
EHR: electronic health record
EU: European Union
GDP: gross domestic product
HIT: health information technology
ICT: information and communication technology
IT: information technology
